# Structure and Anti-Inflammatory Activity of a New Unusual Fucosylated Chondroitin Sulfate from *Cucumaria djakonovi*

**DOI:** 10.3390/md16100389

**Published:** 2018-10-17

**Authors:** Nadezhda E. Ustyuzhanina, Maria I. Bilan, Elena G. Panina, Nadezhda P. Sanamyan, Andrey S. Dmitrenok, Eugenia A. Tsvetkova, Natalia A. Ushakova, Alexander S. Shashkov, Nikolay E. Nifantiev, Anatolii I. Usov

**Affiliations:** 1 N.D. Zelinsky Institute of Organic Chemistry, Russian Academy of Sciences, Leninsky prospect 47, Moscow 119991, Russia; bilan@ioc.ac.ru (M.I.B.); dmt@ioc.ac.ru (A.S.D.); e_tsvet@ioc.ac.ru (E.A.T.);shash@ioc.ac.ru (A.S.S.); nen@ioc.ac.ru (N.E.N.); 2 Kamchatka Branch of Pacific Geographical Institute FEB RAS, Russian Academy of Sciences, Petropavlovsk-Kamchatsky 683000, Russia; panina1968@mail.ru (E.G.P.); actiniaria@sanamyan.com (N.P.S.); 3 V.N. Orekhovich Research Institute of Biomedical Chemistry, Pogodinskaya str. 10, Moscow 119121, Russia; natalia.ushakova@ibmc.msk.ru

**Keywords:** fucosylated chondroitin sulfate, *Cucumaria djakonovi*, sea cucumber, structure, anti-inflammatory activity

## Abstract

Fucosylated chondroitin sulfate **CD** was isolated from the sea cucumber *Cucumaria djakonovi* collected from the Avachinsky Gulf of the eastern coast of Kamchatka. Structural characterization of **CD** was performed using a series of non-destructive NMR spectroscopic procedures. The polysaccharide was shown to contain a chondroitin core [→3)-β-d-GalNAc-(1→4)-β-d-GlcA-(1→]_n_ where about 60% of GlcA residues were 3-O-fucosylated, while another part of GlcA units did not contain any substituents. The presence of unsubstituted both at O-2 and O-3 glucuronic acid residues in a structure of holothurian chondroitin sulfate is unusual and has not been reported previously. Three different fucosyl branches Fuc*p*2*S*4*S*, Fuc*p*3*S*4*S* and Fuc*p*4*S* were found in the ratio of 2:1:1. The GalNAc units were mono- or disulfated at positions 4 and 6. Anti-inflammatory activity of **CD** was assessed on a model of acute peritoneal inflammation in rats. About 45% inhibition was found for **CD**, while a structurally related linear chondroitin sulfate **SS** from cartilage of the fish *Salmo salar* demonstrated only 31% inhibition, indicating that the presence of sulfated fucosyl branches is essential for anti-inflammatory effect of chondroitin sulfates of marine origin.

## 1. Introduction

The body walls of sea cucumbers contain two main types of sulfated polysaccharides, sulfated fucans (SF) often named “fucoidans” [1,2,3,4,5,6], and fucosylated chondroitin sulfates (FucCS). The latter polysaccharides isolated from different species of sea cucumbers are known to be composed of d-glucuronic acid, *N*-acetyl-d-galactosamine, l-fucose and sulfate residues [7]. The backbones of these polysaccharides are formed by repeating disaccharide units →3)-β-d-GalNAc-(1→4)-β-d-GlcA-(1→, while l-fucosyl residues are attached as branches to O-3 of GlcA or O-6 of GalNAc of the backbones. The pattern of sulfation adds the species-to-species structural diversity of FucCS [8]. Thus, sulfate groups could be located at O-4 or both at O-4 and O-6 of GalNAc, at different positions of fucosyl branches, as well as at O-3 or both at O-2 and O-3 of GlcA [9,10,11,12,13,14]. The fine structure of FucCS depends on the species and significantly influences the type and level of biological activity, such as anticoagulant, antithrombotic, antitumor, etc. [15,16]. The anti-inflammatory activity is connected with the ability of FucCS to bind to P- and L-selectins [17]. It should be noted, however, that some data on the distinct correlations between structure of fucose branches and biological activity of FucCS are controversial [18].

About one hundred of holothurian species belonging to 3 subclasses, 5 orders, 16 families and 47 genera were found in the Far-Eastern seas of Russia [19]. Some of these species, such as *Cucumaria djakonovi* (family Cucumariidae, order Dendrochirotida), have potential commercial application as the high-value components of functional foods or the source of biologically active compounds, but chemical composition of the most of them was not investigated. The present communication describes structural characterization of a new type of FucCS isolated from the sea cucumber *C. djakonovi* and designated as **CD**. Anti-inflammatory properties of this polysaccharide were studied as well and compared with the corresponding activity of a linear chondroitin sulfate **SS** isolated from the fish *Salmo salar*.

## 2. Results and Discussion

Water-soluble polysaccharides were isolated from the body walls of *C. djakonovi* by conventional solubilization in the presence of papain [20] followed by treatment of the extract with cetyltrimethylammonium bromide to precipitate the sulfated components, which were then transformed into a water-soluble sodium salts by stirring with NaI in ethanol. The yield of the crude preparation of sulfated polysaccharides (**SP**) was 1.1% of wet body walls. It should be noted that, in addition to Fuc, GalN, GlcA and sulfate as the expected main components, **SP** contained also moderate amounts of Glc, Gal, GlcN and traces of Xyl and Man.

**SP** was, evidently, a mixture of several polysaccharides. It was resolved into four fractions using anion-exchange chromatography on a column containing DEAE(diethylaminoethyl)-Sephacel. Fractions were eluted with water followed by aqueous NaCl solutions of increasing concentrations. According to the molar ratio of the main components (Fuc:GlcA:GalNAc:SO_3_Na, 11:16:16:57), fraction eluted with 1.0 M NaCl may be regarded as a preparation of FucCS, which will be further denoted as **CD**.

A preparation of usual vertebrate chondroitin sulfate **SS** was isolated from a crude extract of *Salmo salar* cartilage [21] using mild alkaline treatment followed by anion-exchange chromatography. Structure of **SS** was confirmed by coinciding of its NMR spectral characteristics with literature data for chondroitin sulfates A and C [21,22,23]. According to NMR spectra, the ratio between A and C units in **SS** was ~0.8 (see Figures S1–S4).

**Figure 1:**
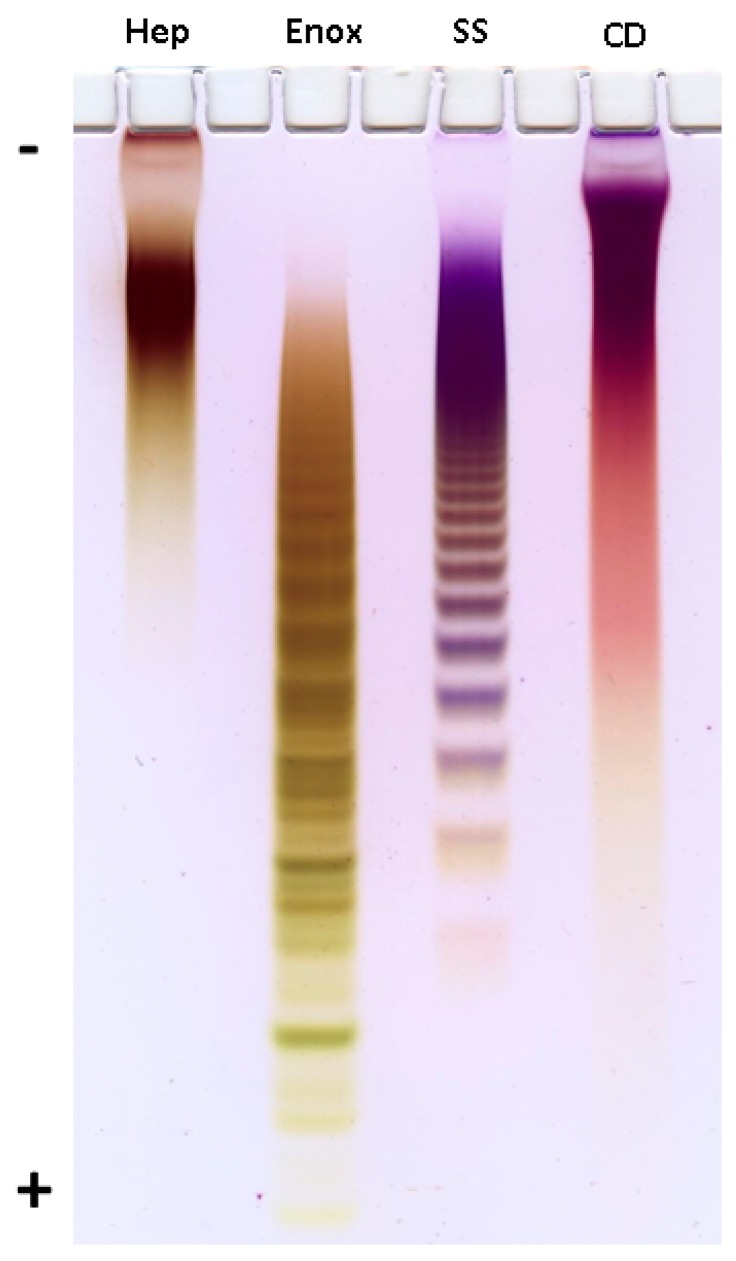
Electrophoresis in polyacrylamide gel. Hep—heparin (Sigma), Enox—enoxaparin (Clexane^®^, Sanofi), **SS**—chondroitin sulfate from *S. salar*, **CD**—fucosylated chondroitin sulfate from *C. djakonovi*.

Preliminary assessment of the molecular weights of **CD** and **SS** was performed by polyacrylamide gel electrophoresis (PAGE) in comparison with sulfated polysaccharides heparin (Sigma, St. Louis, MO, USA) and enoxaparin (Clexane^®^, Sanofi, Paris, France) having defined molecular weight (MW) and used as standards (Figure 1). Thus, MW of heparin and enoxaparin were stated to be about 17 kDa and 4.5 kDa, respectively, and the difference in MW was definitely seen on PAGE. Based on mobility of samples it was concluded that MW of **CD** was quite similar to that of heparin. More accurate estimation of MW was performed by TSK gel chromatography using an appropriate analytical column calibrated with pullulans. As a result, the molecular weight of **CD** was determined as ~17.3 kDa.

**Figure 2:**
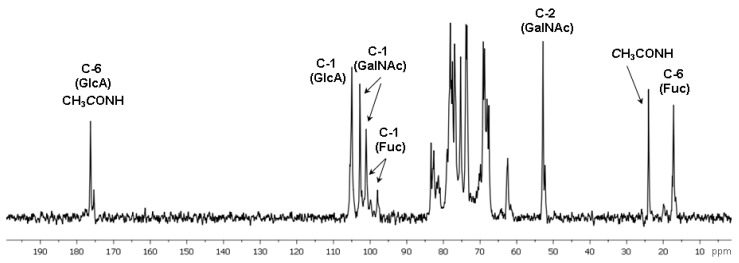
The ^13^C NMR spectrum of fucosylated chondroitin sulfate **CD**.

**Figure 3:**
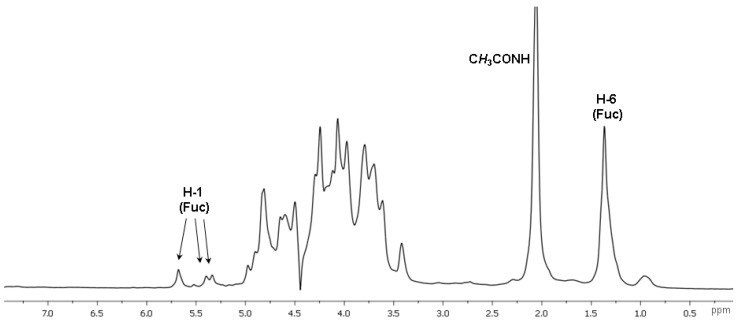
The ^1^H NMR spectrum of fucosylated chondroitin sulfate **CD**.

Further detailed characterization of the structure of polysaccharide **CD** was performed using a series of non-destructive NMR spectroscopic procedures. Analysis of the 1D NMR spectra confirmed the presence of N-acetyl-galactosamine, uronic acid and fucose residues as the main monosaccharide components by the characteristic values of chemical shifts of C-2 (*δ* 52.7 ppm) for GalNAc, and C-6 for Fuc (*δ* 16.9, 17.2 ppm) and GlcA (*δ* 175.6–176.0 ppm) in the ^13^C NMR spectrum, as well as of H-6 (*δ* 1.37 ppm) for Fuc in the ^1^H NMR spectrum (Figure 2 and Figure 3, Table 1).

**Figure 4:**
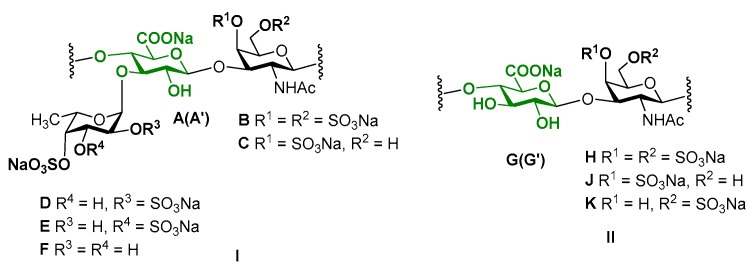
Repeating branched (**I**) and linear (**II**) blocks of fucosylated chondroitin sulfate **CD** (units **A**–**K**) and disaccharide components of chondroitin sulfate **SS** (units **G, G’, J, K**). Unit **A** bears Fuc2*S*4*S* (**D**), whereas unit **A’** bears Fuc3*S*4*S* (**E**) or Fuc4*S* (**F**). Unit **G** is linked to GalNAc4*S*6*S* (**H**) or GalNAc4*S* (**J**), whereas unit **G’** is linked to GalNAc6*S* (**K**).

**Figure 5:**
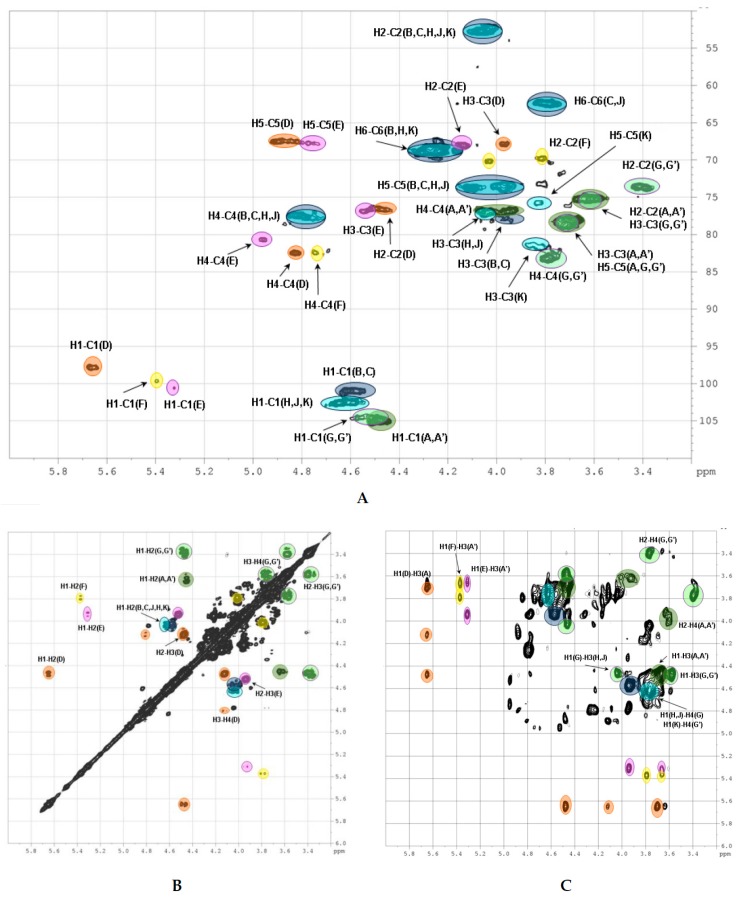
The NMR spectra of fucosylated chondroitin sulfate **CD**: ^1^H-^13^C HSQC (**A**), ^1^H-^1^H COSY (**B**), ROESY (**C**). Correlation peaks are marked in the spectra by letter designation of units used in Figure 4 and Table 1.

The 2D NMR experiments led to full assignment of all the signals in ^1^H and ^13^C NMR spectra and to reveal the fine structure of polysaccharide **CD** (Figure 4). There were six separated cross-peaks H1/C1 in the anomeric region of the ^1^H-^13^C HSQC NMR spectrum of **CD** (Figure 5A). Three of them at 5.69/97.7 ppm, 5.34/100.5 ppm and 5.41/99.6 ppm were related to fucosyl residues Fuc*p*2*S*4*S* (**D**), Fuc*p*3*S*4*S* (**E**) and Fuc*p*4*S* (**F**), respectively [9,10,11,12,13,14,17]. The assignment of signals in the spin-systems of units **D**–**F** was performed based on the data of the COSY, ROESY (Figure 5B, C), and TOCSY (Figure S4) spectra (Table 1). The downfield chemical shifts of the respective protons and carbons confirmed the pattern of sulfation of units **D**–**F**. The attachment of all fucosyl branches to O-3 of GlcA residues was confirmed by the correlation peaks H-1(Fuc)-H-3(GlcA) in the ROESY spectrum (Figure 5C). The ratio of **D**:**E**:**F** was determined using the integral intensities of the respective H-1 signals and was found to be 2:1:1. These data were consistent with those obtained previously for many other FucCS bearing sulfated fucosyl branches [17,24,25,26,27,28,29,30].

Two other cross-peaks H_1_/C_1_ 4.47/105.1 ppm and 4.58/100.9 ppm in the ^1^H-^13^C HSQC spectrum are related to the units GlcA (**A**, **A’**) and GalNAc (**B**, **C**), respectively, which are typical components of a backbone [→3)-β-d-GalNAc-(1→4)-β-d-GlcA-(1→]_n_ of fucosylated chondroitin sulfates [9,10,11,12,13,14,17]. The assignment of ^1^H signals in the spin-systems of units **A**, **A’**, **B** and **C** was performed based on the data of COSY, ROESY (Figure 5B,C), and TOCSY (Figure S5) spectra (Table 1). The respective ^13^C signals were easily determined using the HSQC spectrum.

The unusual cross-peak H_1_/C_1_ (4.56–4.59)/(102.4–102.6) ppm in the HSQC spectrum was found to be related to the GalNAc units (**H**, **J**, **K**). Thus, the respective correlation H_1_/H_2_ in the COSY spectrum indicated the position of H_2_ at 4.03 ppm, which is typical for the GalNAc units (Figure 5B). Moreover, the COSY spectrum led to reveal another type of the GlcA units in a structure of **CD**. Besides units **A** and **A’** described above, there were residues **G** and **G’** having H_1_ and C_1_ signals overlapped with those of **A** and **A’**. As the result, the seventh cross-peak H_1_/C_1_ related to G/G’ residues was masked in the HSQC spectrum. Fortunately, the values of chemical shifts of other signals of **G** (**G’**) were significantly different, indicating the distinction in substitution of units **A** (**A’**) and **G** (**G’**) (Table 1). The high field shifts of C_2_ (73.9 ppm) and C_3_ (75.2 pm) signals pointed the lack of any substituents in these positions, while the low field shift of C_4_ signal (81.8 and 82.9 ppm) supported the presence of (1→4)-linkage. The connections between GalNAc units and GlcA units was determined by the cross-peaks H_1_(GalNAc)-H_4_(GlcA) and H_1_(GlcA)-H_3_(GalNAc) in the ROESY spectrum (Figure 5C). Therefore, linear fragments composed of the disaccharides →3)-β-d-GalNAc4*S*6*S*-(1→4)-β-d-GlcA-(1→, →3)-β-d-GalNAc4*S*-(1→4)-β-d-GlcA-(1→, and →3)-β-d-GalNAc6*S*-(1→4)-β-d-GlcA-(1→ were found in **CD**. To confirm the presence of unsubstituted both at O-2 and O-3 glucuronic acid residues in the structure of **CD** we compared its spectra with those of chondroitin sulfate **SS** bearing such structural motive (Figure 2 and Figures S1–S4). Coincidence of the respective signals successfully evidenced the presence of linear fragments in a structure of **CD**. The ratio of the linear and branched blocks was calculated using the values of integral intensity of the H1/C1 cross-peaks of units (**B**, **C**) and (**H**, **J**, **K**) in the HSQC NMR spectrum and was found to be about 2:3.

Chondroitin sulfates are known as complex molecules demonstrating a wide range of biological activities [31], including anti-inflammatory action [32]. Previously several chondroitin sulfates obtained from fish cartilages were studied as anti-inflammatory agents, using a model of acute peritoneal inflammation in rats [21]. It was interesting to compare anti-inflammatory properties of typical vertebrate chondroitin sulfate and fucosylated chondroitin sulfate from sea cucumber. For this purpose, purified chondroitin sulfate **SS** was prepared from cartilage of the fish *S. salar.* Two polysaccharides **CD** and **SS** were studied as anti-inflammatory agents in vivo on a model of acute peritoneal inflammation in rats. The acute inflammatory response is characterized by tissue influx of neutrophilic granulocytes (neutrophils), which are considered as main effectors at the beginning stage [33,34]. The number of neutrophils in the exudate was calculated after 3 h of inflammation (Table 2). About 45% inhibition was found for **CD**, while structurally related chondroitin sulfate **SS** demonstrated only 31% inhibition indicating that structural features of **CD**, primarily the presence of fucosyl branches and greater degree of sulfation, are essential for more pronounced anti-inflammatory effect. More detailed immunological study of the anti-inflammatory effect of FCS will be described further with the use of a series of structurally different polysaccharides from several sea cucumber species.

## 3. Materials and Methods

### 3.1. General Methods

Quantitative determination of monosaccharides by hydrolysis in 2 M CF_3_COOH at 100 °C for 8 h followed by transformation into alditol acetates and gas-liquid chromatography, as well as turbidimetric determination of sulfate were carried out as described previously [35,36]. For determination of hexosamines acid hydrolysis in 6 N HCl at 100 °C for 6 h was used. Glucuronic acid was estimated colorimetrically with 3,5-dimethylphenol [37]. Molecular weight of the polysaccharide was estimated by gel chromatography on analytical TSK4000SW_XL_ column (Toyo Soda, Japan, 7.5 × 300 mm) calibrated using pullulans (Fluka) and eluted with 1 M NaCl.

### 3.2. Isolation of Polysaccharide **CD**

The sea cucumber *Cucumaria djakonovi* (Baranova, 1980), family Cucumariidae, order Dendrochirotida, was collected by diving on 14 September 2016 from Kamchatka coastal waters (Avachinsky Gulf near Starichkov Island, at 52.46°203′ N, 158.37°362′ E, depth 20 m) and fixed with ethanol. The specimen was identified by one of the authors (E.G.Panina), preparation No. 759 is deposited at the Kamchatka Branch of Pacific Geographical Institute FEB RAS, Petropavlovsk-Kamchatsky 683000, Russia. According to the conventional procedure [20], the wet body walls (19.3 g) were minced, suspended in 200 mL of 0.1 M sodium acetate buffer pH 6.0 containing papain (0.7 g), EDTA (0.3 g) and l-cysteine hydrochloride (0.2 g), and incubated at 45–50 °C for 24 h. An aqueous solution of hexadecyltrimetylammonium bromide (10%, 60 mL) was added to the filtered extract, the resulting precipitate was stirred with 20% ethanolic NaI solution (5 × 50 mL) for 2–3 days, washed with ethanol, dissolved in water, filtered and lyophilized to give crude polysaccharide preparation **SP**, yield 219 mg, composition: Fucose, 8.0%, galactosamine, 5.7%, uronic acids, 5.5%, galactose, 5.8%, glucose, 3.0%, glucosamine, 1.9%, and sulfate, 28.3%.

A solution of **SP** (200 mg) in water (50 mL) was placed on a column (3 × 10 cm) with DEAE-Sephacel in Cl^−^-form and eluted with water, followed by NaCl solutions of increasing concentration (0.5, 0.75, 1.0 and 1.5 M), each time up to the absence of a positive reaction of eluate for carbohydrate [38]. Eluate at 1.0 M NaCl was desalted on Sephadex G-15 and lyophilized giving rise to preparation **CD** (56 mg). The polysaccharide contained Fuc, GalNAc, GlcA and sulfate in a molar ratio of 11:16:16:57 with negligible content of other monosaccharides.

### 3.3. Purification of Polysaccharide **SS**

A crude extract from cartilage of *Salmo salar* [21] (100 mg) was dissolved in water (2 mL) and 1 M aq NaOH solution (0.1 mL) was added. The mixture was kept for 3 h at 40 °C. Then the solution was neutralized by 0.1 M HCl and placed on a column containing DEAE-Sephacel in Cl^−^-form. The column was eluted with water followed by NaCl solutions of increasing concentration (0.5, 0.75, 1.0 and 1.5 M), each time up to the absence of a positive reaction of eluate for carbohydrate [38]. Eluate at 1.0 M NaCl was desalted on Sephadex G-15 column and lyophilized to give polysaccharide **SS** (40 mg).

### 3.4. Polyacrylamide Gel Electrophoresis (PAGE)

The polysaccharides **CD**, **SS**, heparin (Sigma-Aldrich, St. Louis, MO, USA) and enoxaparin (Clexane^®^, Sanofi, Paris, France) (15 μg) were applied to a 0.75-mm-thick layer of 10% polyacrylamide (ICN Biochemicals), 100 mM Tris-borate, pH 8.3 gel in a buffer (10 mM Tris-borate, pH 8.3) with 10% (*w*/*v*) of glycerol. Electrophoresis was run at 400 V in a buffer (100 mM Tris-borate, pH 8.3) during 1 h. The gel was stained with 0.003% Stains-all (Merck, DE) in formamide (Sigma, EUA)—isopropanol—water (5:25:70) overnight in the dark and destained with water. The results are presented on Figure 1.

### 3.5. NMR Spectroscopy

The sample **CD** (30 mg) were dissolved in 99.9% D_2_O, freeze-dried, dissolved in 99.96% D_2_O and put into Shigemi tube. The value of pH was adjusted to 7.0 by addition of 0.01 M NaOD solution in D_2_O. The 1D and 2D NMR spectra of the samples in D_2_O were recorded on a 600 MHz Avance II (Bruker, Germany) NMR spectrometer equipped with a z-gradient probe with proton and carbon frequencies of 600.13 and 150.90 MHz respectively. 3-(Trimethylsilyl)-2,2,3,3-tetradeuteropropionic acid (TSP) was used as an internal standard (δ_H_ 0.0 ppm, δ_C_ −1.6 ppm). The conditions of the experiments were described previously [12].

### 3.6. Model of Acute Peritoneal Inflammation in Rats

The animal protocols used in this work were evaluated and approved by the local ethical committee of the V.N. Orekhovich Research Institute of Biomedical Chemistry (Protocol 04-2018). They are in accordance with the order 490 (5 November 2008) of the Agricultural Ministry of Russian Federation, meet National GLP Standard of Russian Federation (53434-2009).

The peritoneal inflammation in rats was performed according to a method described earlier [39,40]. A 9.0% solution of peptone in 0.9% NaCl (8 mL) was administered intraperitoneally to female Wistar rats (ca. 250 g) under ether narcosis. Solutions of polysaccharides CD and SS (1 mg in 0.3 mL of sterile 0.9% NaCl) were administered to a rat femoral vein under ether narcosis 15 min after the injection of peptone. Sterile 0.9% NaCl (0.3 mL) was administered intravenously to control animals. After 3 h, the animals were decapitated under ether narcosis. The abdominal cavity was washed with a medium (30 mL) containing PBS, heparin (60 unit/mL), 0.02% EDTA, and 0.03% bovine serum with intense peritoneal massage. The total cell number in the washing liquid was counted in Goryaev’s chamber. To calculate the number of neutrophils, the cell suspension was centrifuged at 400 g for 10 min. The concentrated suspension was diluted with a whole bovine serum (1:1). Smears were made and stained by the May Grünwald Giemsa method. The percentage of neutrophils in the smears was determined by counting of 6–8 hundred cells using a double blind method. The total number of neutrophils in the exudate was calculated from the percentage of neutrophils and the total cell number. Data in the group were presented in the format of mean and standard deviation (Mean ± SD). Analysis of the reliability of differences was carried out using the t-criterion. Differences were considered significant at *p* < 0.05.

## 4. Conclusions

Fucosylated chondroitin sulfates (FucCS) are specific components of the holothurian body walls. Structural analysis of FucCS is mainly based on the data of NMR spectroscopy [23,41,42]. Fine structures of FucCS depend on the sea cucumber species [7,8]. Isolation and structural analysis of new representatives of FucCS give new evidence on the structural diversity of this important class of sulfated glycosaminoglycans. Thus, a new polysaccharide (**CD**) was found in *Cucumaria djakonovi*, the holothurian species collected from Kamchatka coastal waters. As followed unambiguously from the NMR spectra of **CD**, the polysaccharide contained, side by side with usual repeating multiple sulfated trisaccharide units α-l-Fuc-(1→3)-β-d-GlcA-(1→3)-β-d-GalNAc, also substantial amount of disaccharide repeating units β-d-GlcA-(1→3)-β-d-GalNAc sulfated at O-4, O-6 or both at O-4 and O-6 of GalNAc (units of chondroitin sulfates A, C and E, respectively), typical of chondroitin sulfates (CS) of vertebrates, where GlcA residues bear no substituents both at O-2 and O-3. Among various biological activities described for CS and FucCS, marked anti-inflammatory action of these polysaccharides was found and explained by their possible interaction with P- and L-selectins [17,21,43]. Comparison of anti-inflammatory activity of **CD** and **SS** (a linear CS isolated from the fish *Salmo salar* cartilage) made it possible to demonstrate the enhanced biological activity of molecules bearing sulfated fucosyl branches.

## Figures and Tables

**Table 1:** The data of ^1^H and ^13^C NMR spectra of fucosylated chondroitin sulfate **CD** and chondroitin sulfate **SS** (the bold numerals indicate the positions of sulfate). The residues are designated as in Figure 4.

Residue	H1/C1	H2/C2	H3/C3	H4/C4	H5/C5	H6/C6
**A** →4)-β-d-Glc*p*A-(1→	4.48/105.0	3.64/75.0	3.71/78.1	3.96/76.6	3.71/78.1	- 176.0
**A’** →4)-β-d-Glc*p*A-(1→	4.48/105.0	3.60/75.0	3.68/80.7	4.00/76.6	3.71/78.1	- 176.0
**B** →3)-β-d-Gal*p*NAc4*S*6*S*-(1→	4.58/100.9	4.07/52.7	3.95/77.9	**4.81**/**77.2**	4.00/73.2	**4.33**, **4.20** **68.5**
**С** →3)-β-d-Gal*p*NAc4*S*-(1→	4.58/100.9	4.07/52.7	3.95/77.9	**4.81**/**77.2**	4.02/76.2	3.81/62.3
**D** α-l-Fuc*p*2*S*4*S*-(1→	5.69/97.7	**4.48**/**76.6**	4.17/67.8	**4.86**/**82.5**	4.90/67.5	1.37/16.9
**E** α-l-Fuc*p*3*S*4*S*-(1→	5.34/100.5	3.95/67.6	**4.53**/**76.6**	**5.01**/**80.6**	4.80/67.6	1.37/17.2
**F** α-l-Fuc*p*4*S*-(1→	5.41/99.6	3.82/69.7	4.04/70.0	4.77/82.4	4.80/67.6	1.37/17.2
**G** →4)-β-d-Glc*p*A-(1→	4.47/105.1	3.38/73.9	3.59/75.2	3.78/81.8	3.70/77.9	- 175.9
**G’** →4)-β-d-Glc*p*A-(1→	4.50/105.3	3.38/73.9	3.59/75.2	3.74/82.9	3.70/77.9	- 175.7
**H** →3)-β-d-Gal*p*NAc4*S*6*S*-(1→	4.59/102.4	4.03/52.8	4.03/76.8	**4.75**/**77.6**	4.12/74.0	**4.24**/**68.9**
**J** →3)-β-d-Gal*p*NAc4*S*-(1→	4.59/102.4	4.03/52.8	4.03/76.8	**4.75**/**77.6**	3.83/75.8	3.80/62.3
**K** →3)-β-d-Gal*p*NAc6*S*-(1→	4.56/102.6	4.03/52.3	3.86/81.5	4.18/68.9	3.98/74.0	**4.24**/**68.9**

**Table 2:** Anti-inflammatory activity of the polysaccharides **SS** and **CD**.

Sample	Number of Animals	Number of Neutrophils (×10^6^)	p	% of Inhibition
Control	6	69.4 ± 6.4		-
**SS**	4	47.8 ± 5.2	<0.002	31.2
**CD**	4	38.2 ± 4.9	<0.002	45.0

## Supplementary Materials

The following are available online at http://www.mdpi.com/1660-3397/16/10/389/s1, Figure S1: The ^1^H NMR (**A**) and ^13^C NMR (**B**) spectra of chondroitin sulfate **SS**, Figure S2: The ^1^H-^13^C HSQC NMR spectrum of chondroitin sulfate **SS**, Figure S3: The ^1^H-^1^H COSY NMR spectrum of chondroitin sulfate **SS**, Figure S4: The HMBC spectra of chondroitin sulfate **SS** (**A**) and fucosylated chondroitin sulfate **CD** (**B**). Figure S5: The ^1^H-^1^H TOCSY NMR spectrum of fucosylated chondroitin sulfate **CD**.
