# Supplementary material for: Structure and Anti-Inflammatory Activity of a New Unusual Fucosylated Chondroitin Sulfate from Cucumaria djakonovi
Source: Mar Drugs. 2018 Oct 17;16(10):389. doi: 10.3390/md16100389 (PMC6212937; doi:10.3390/md16100389)
Supplement: Supplementary file 1 [file marinedrugs-16-00389-s001.pdf]

Supplementary information

Structure and Anti-Inflammatory Activity of a New Unusual Fucosylated Chondroitin Sulfate from *Cucumaria djakonovi*

Nadezhda E. Ustyuzhanina [1,*], Maria I. Bilan [1], Elena G. Panina [2], Nadezhda P. Sanamyan [2], Andrey S. Dmitrenok [1], Eugenia A. Tsvetkova [1], Natalia A. Ushakova [3], Alexander S. Shashkov [1], Nikolay E. Nifantiev [1] and Anatolii I. Usov [1,*]

[1] N.D. Zelinsky Institute of Organic Chemistry, Russian Academy of Sciences, Leninsky prospect 47, Moscow 119991, Russia; bilan@ioc.ac.ru (M.I.B.); dmt@ioc.ac.ru (A.S.D.); e_tsvet@ioc.ac.ru (E.A.T.); shash@ioc.ac.ru (A.S.S.); nen@ioc.ac.ru (N.E.N.)

[2] Kamchatka Branch of Pacific Geographical Institute FEB RAS, Russian Academy of Sciences, Petropavlovsk-Kamchatsky 683000, Russia; panina1968@mail.ru (E.G.P.); actiniaria@sanamyan.com (N.P.S.)

[3] V.N. Orekhovich Research Institute of Biomedical Chemistry, Pogodinskaya str. 10, Moscow 119121, Russia; natalia.ushakova@ibmc.msk.ru

* Correspondence: ustnad@gmail.com (N.E.U.); usov@ioc.ac.ru (A.I.U.); Tel.: +7-495-135-8784 (N.E.U.)

A

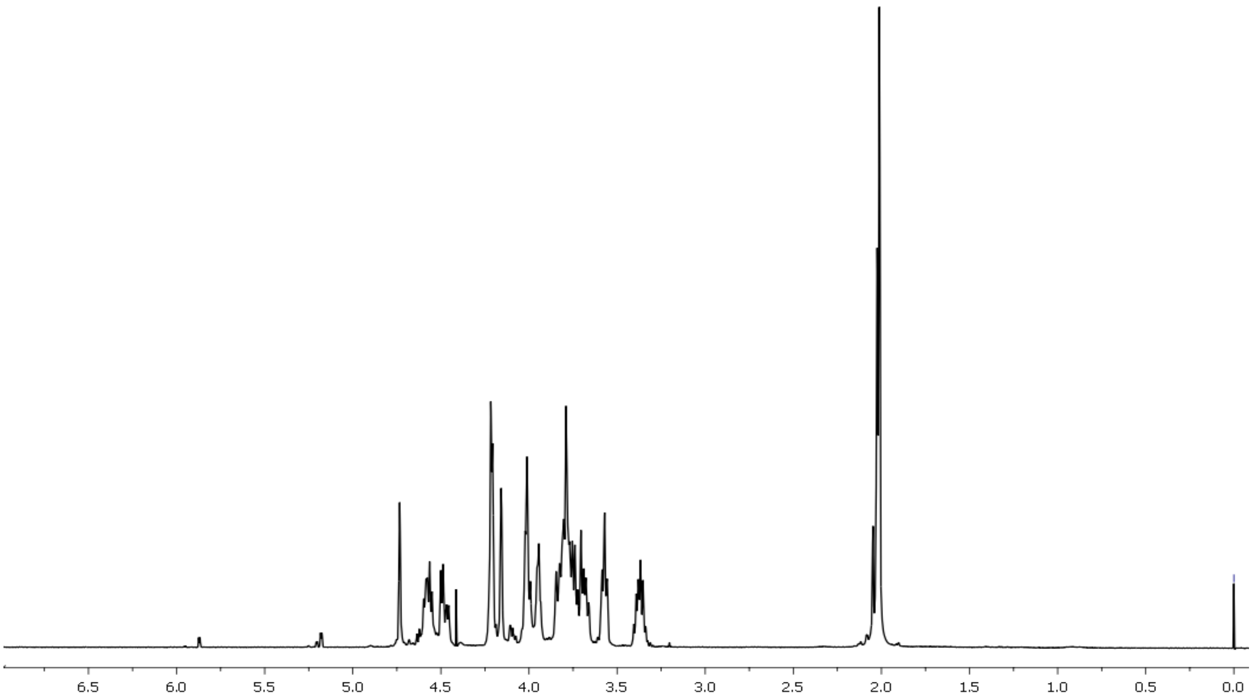

B

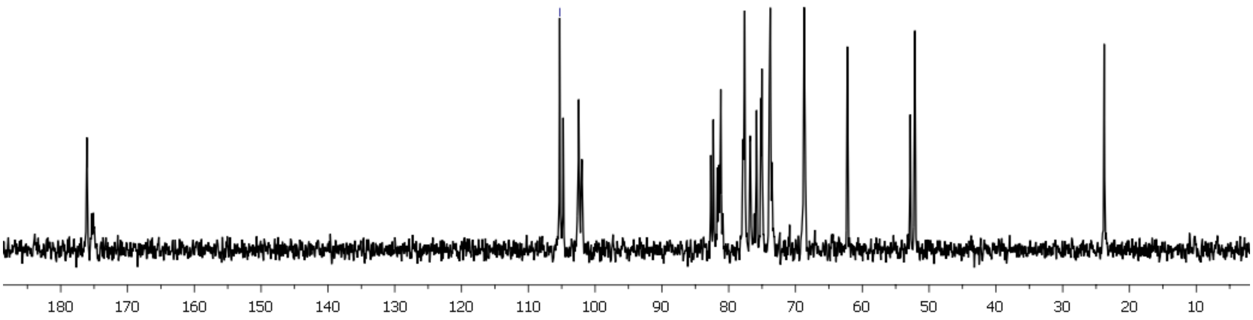

Figure S1. The ^{1}H NMR (**A**) and ^{13}C NMR (**B**) spectra of chondroitin sulfate **SS**.

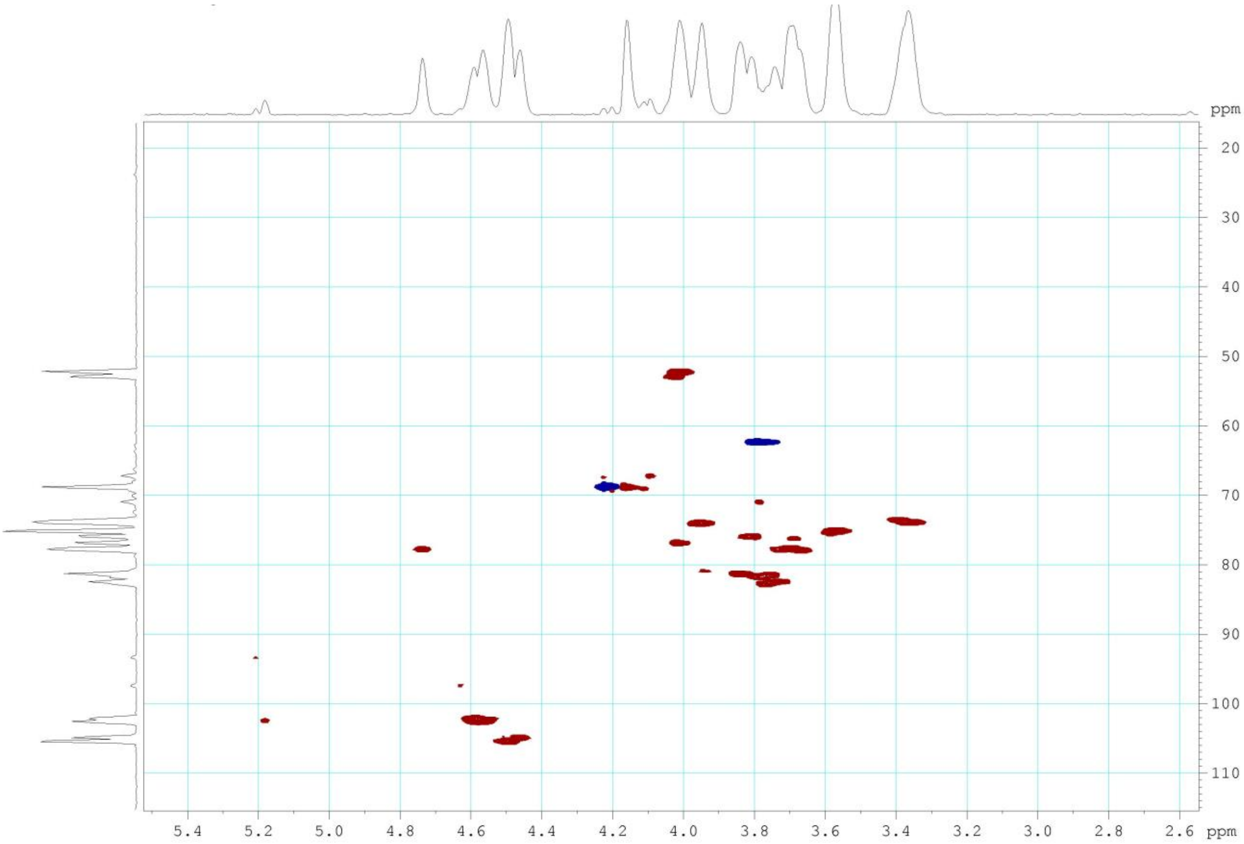

Figure S2. The ^{1}H-^{13}C HSQC NMR spectrum of chondroitin sulfate **SS**.

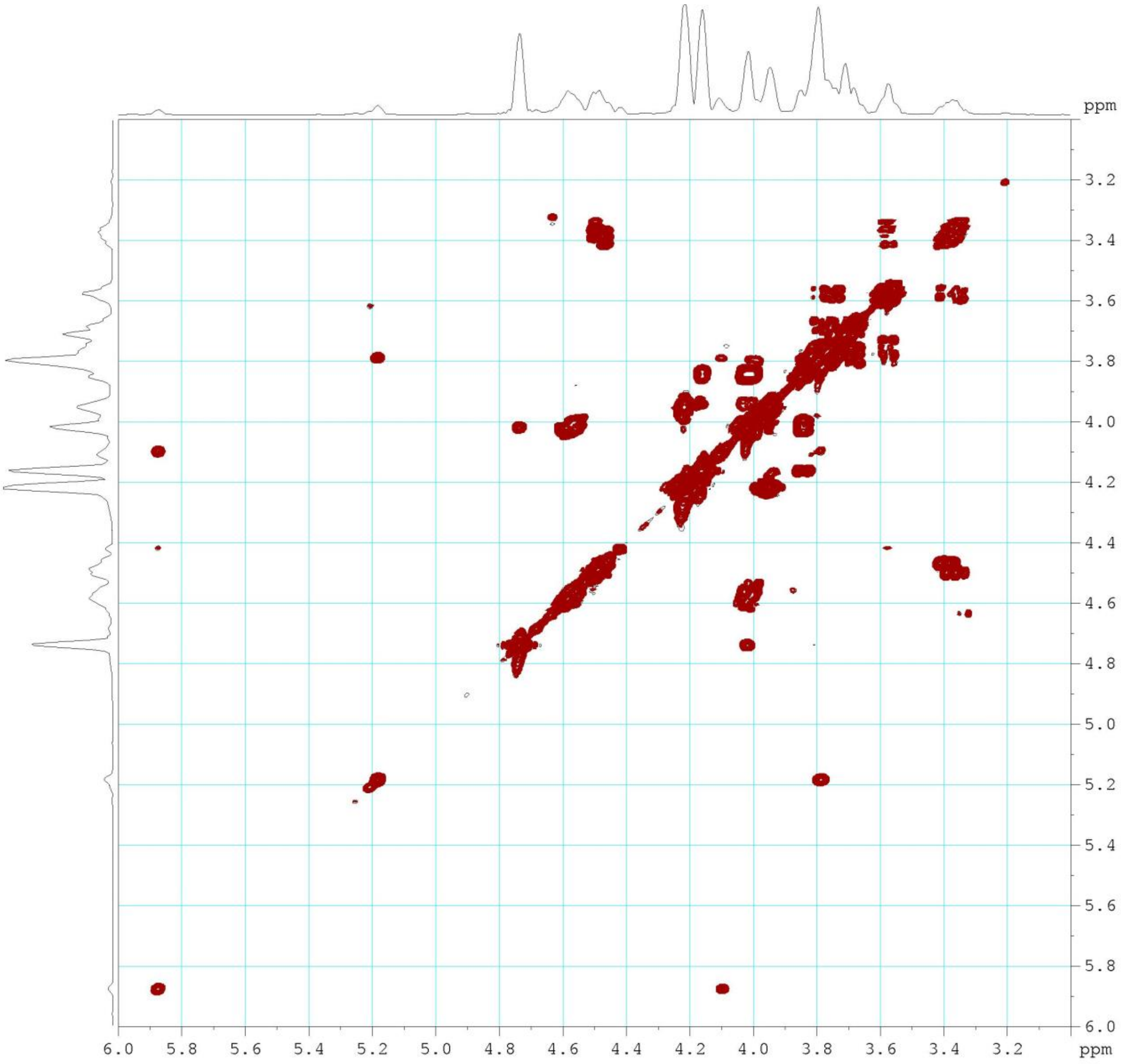

Figure S3. The ^1H-^1H COSY NMR spectrum of chondroitin sulfate **SS**.

A

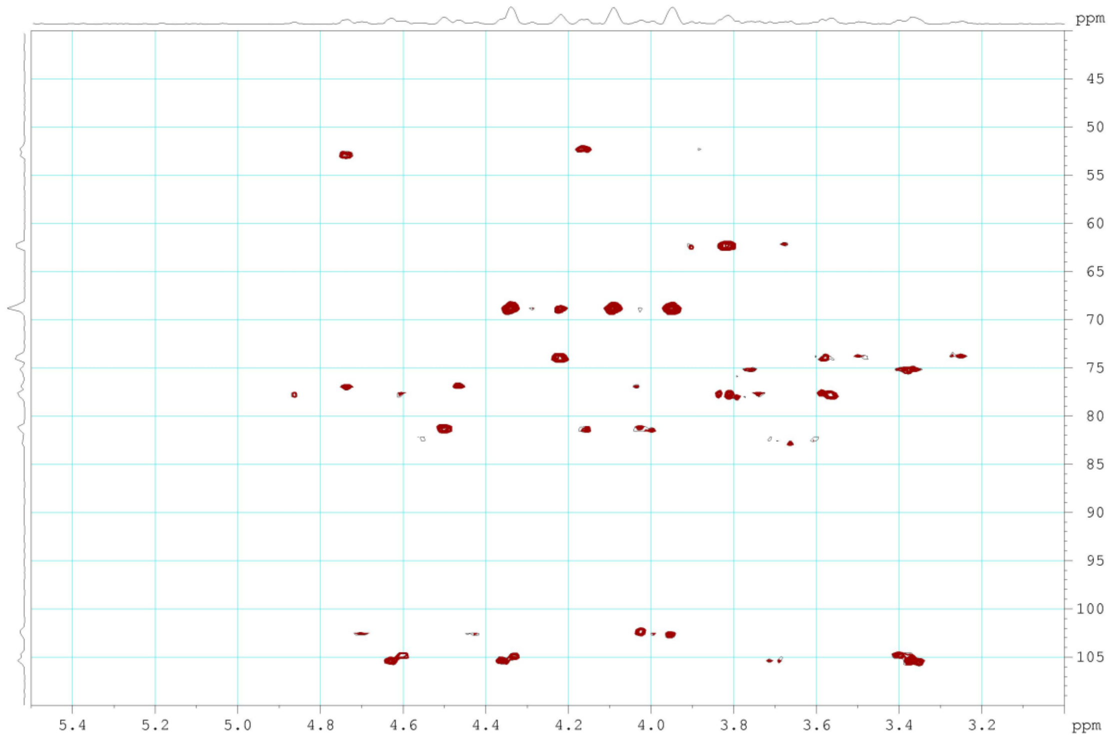

B

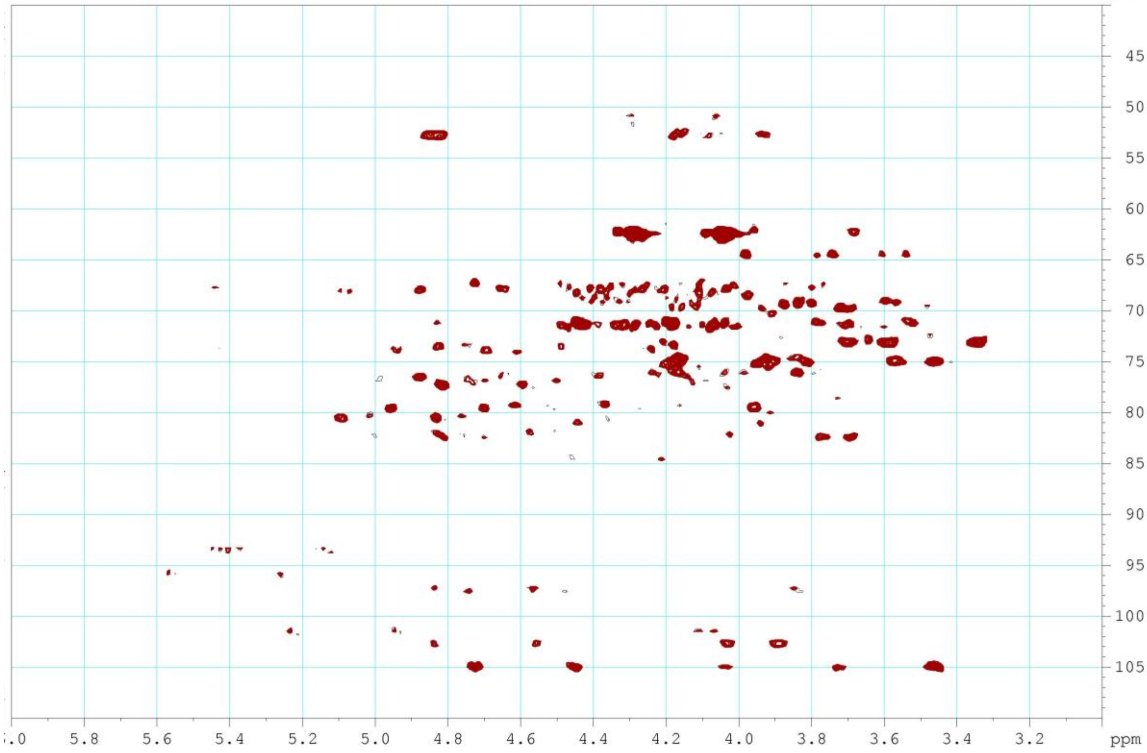

Figure S4. The HMBC spectra of chondroitin sulfate **SS** (**A**) and fucosylated chondroitin sulfate **CD** (**B**).

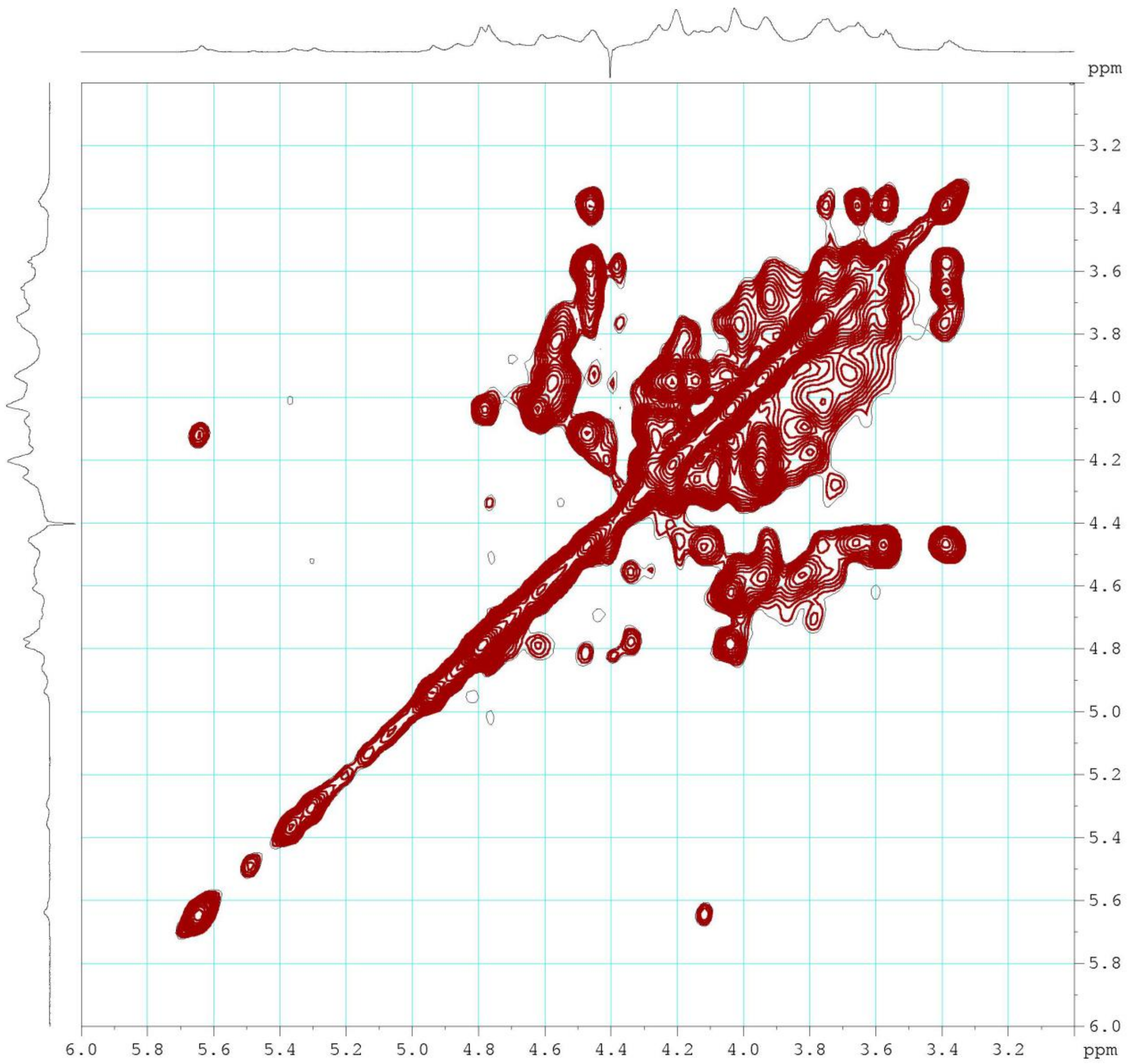

Figure S5. The ^{1}H-^{1}H TOCSY spectrum of fucosylated chondroitin sulfate **CD**.
